# The Ongoing Challenges of Hearing Loss: Stigma, Socio-Cultural Differences, and Accessibility Barriers

**DOI:** 10.3390/audiolres15030046

**Published:** 2025-04-24

**Authors:** Mirko Aldè, Umberto Ambrosetti, Stefania Barozzi, Samantha Aldè

**Affiliations:** 1 Department of Clinical Sciences and Community Health, University of Milan, 20122 Milan, Italy; umberto.ambrosetti@unimi.it (U.A.); stefania.barozzi@unimi.it (S.B.); 2 Audiology Unit, Department of Specialist Surgical Sciences, Fondazione IRCCS Ca’ Granda Ospedale Maggiore Policlinico, 20122 Milan, Italy; 3 Department of Sociology and Social Research, University of Milano-Bicocca, 20126 Milan, Italy; s.alde1@campus.unimib.it

**Keywords:** hearing loss, deafness, accessibility, stigma, assistive technologies

## Abstract

**Background/Objectives**: Hearing loss (HL) is a prevalent condition that can lead to social exclusion. This review explores the epidemiological, cultural, and social dimensions of HL and examines the barriers to accessibility that individuals with HL encounter. **Methods**: This research employs a narrative review approach to provide a comprehensive overview of HL, focusing on stigma, gender disparities, cultural and social differences, and accessibility challenges. **Results**: The review highlights pervasive prejudices surrounding HL and hearing devices. Gender disparities are evident, with Deaf women facing compounded challenges. Cultural perspectives on HL differ widely, ranging from the medical model, which emphasizes treatment using hearing devices, to the Deaf community’s social model, which views deafness as a cultural identity. Socioeconomic disparities further restrict access to modern technologies, particularly in low-income settings, while intersectional discrimination affects marginalized groups within the Deaf community. Public spaces also present significant barriers related to communication, architecture, and technology, which hinder accessibility for individuals with HL. **Conclusions**: A cultural shift is essential to dismantle societal stereotypes and reduce discrimination associated with HL. Moreover, improving accessibility for individuals with HL necessitates a multifaceted approach, including accessible design, staff training, and the integration of assistive technologies.

## 1. Introduction

Hearing loss (HL) remains a significant yet often overlooked condition that affects a large portion of the global population. Despite its prevalence, it is frequently perceived as an “invisible” disability, leading to misunderstandings, social barriers, and exclusion from various aspects of life [1]. Prejudices surrounding Deafness have historically hindered the full participation of individuals with HL in many experiences, limiting their access to education, employment, culture, and social interaction [2]. The lack of awareness about the specific needs of people with HL continues to pose challenges, making it crucial to promote educational campaigns, raise the public consciousness, and implement inclusive initiatives. Accessibility plays a fundamental role in breaking down physical, social, and psychological barriers that contribute to the marginalization of individuals with HL. Ensuring accessibility is not merely about providing hearing devices; it is about fostering an environment where communication is facilitated, diversity is valued, and equal opportunities are guaranteed [3]. The implementation of inclusive policies, assistive technologies, captioning, and sign language interpretation services can significantly enhance independence, self-esteem, and overall well-being for people with HL. In this context, improving accessibility should not be seen as an isolated initiative but as a broader commitment to social equity and inclusion. When institutions, organizations, and communities invest in accessible services and infrastructures, they not only empower individuals with HL but also contribute to a more cohesive and enriched society [4]. Moreover, recognizing the economic potential of improving accessibility is essential. By addressing the needs of individuals with HL, businesses and public services can reach a wider audience and promote sustainable development [5].

This narrative review aims to explore the complex relationship between HL, prejudice, and accessibility by analyzing the challenges and opportunities faced by individuals with hearing impairments. Specifically, it examines key aspects of HL, including its definition, classification, causes, epidemiology, and impact on quality of life, as well as the available treatments. A significant focus is placed on the psychological, social, cultural, and gender-related aspects of HL, with particular attention to social stigma, gender differences, and cultural perspectives. Furthermore, the research delves into the intersection between HL and accessibility, highlighting the barriers that individuals with hearing impairments encounter and evaluating strategies to promote inclusion. By providing a comprehensive overview of these issues, this study seeks to raise awareness, dispel misconceptions, and contribute to the development of a more equitable and inclusive society where accessibility is recognized as a fundamental right.

## 2. Hearing Loss

### 2.1. Definition and Classifications

Hearing loss (HL) is a condition characterized by a reduction in an individual’s auditory capacity, which can vary in type and severity [6]. HL present at birth is classified as “congenital”, whereas HL that develops later is termed “acquired”. Specifically, HL occurring before speech and language development is considered “pre-lingual”, while HL that arises after speech and language acquisition is defined as “post-lingual”. HL can affect only one ear (“unilateral HL”) or both ears (“bilateral HL”) and may either worsen over time (“progressive HL”) or remain stable (“non-progressive HL”) [7]. Based on the location of the impairment, HL is further classified as conductive, sensorineural, or mixed.

*Conductive HL* results from alterations in the structures that transmit sound from the external ear (auricle and external auditory canal) and/or middle ear (tympanic membrane and ossicular chain) to the inner ear. The main causes of conductive HL include the obstruction of the external auditory canal (e.g., due to earwax, foreign bodies, or external otitis), Eustachian tube dysfunction (which may lead to mucus accumulation in the middle ear, as seen in otitis media with effusion), tympanic membrane perforations (e.g., resulting from acute otitis media), otosclerosis (a condition restricting the movement of the stapes), congenital malformations such as aural atresia, and, more rarely, neoplasms. Conductive HL is generally a “mechanical” problem in which the function of the inner ear remains intact, leading to mild to moderate hearing impairment [8,9].

*Sensorineural HL* results from damage to either the hair cells of the cochlea (“cochlear HL”) or the auditory pathways beyond the cochlea (“retrocochlear HL”), including the auditory nerve and, less commonly, structures on the way up to the auditory cortex. The most common cause of sensorineural HL is genetic, accounting for approximately 50–60% of cases. Genetic HL is classified as “non-syndromic” (about 80% of cases) when it presents as an isolated condition and “syndromic” (about 20% of cases) when it occurs alongside other signs and symptoms. Non-syndromic genetic HL can be further categorized based on inheritance patterns as autosomal recessive (about 75–80% of cases), autosomal dominant (about 20% of cases), X-linked (about 2–5% of cases), or mitochondrial (about 1% of cases) [7]. In approximately 40–50% of cases, sensorineural HL is attributed to environmental factors, including exposure to high-intensity noise (“noise-induced HL”), viral infections (e.g., congenital cytomegalovirus infection), congenital malformations of the inner ear (e.g., enlarged vestibular aqueduct and Mondini malformation), cranial trauma, ototoxic drug exposure (e.g., aminoglycoside antibiotics), Ménière’s disease, birth asphyxia, prematurity, and aging (“presbycusis”) [10].

*Mixed HL* is characterized by the coexistence of conductive and sensorineural hearing impairments. Its causes may include cranial trauma, severe infections (such as cholesteatomatous otitis media and meningitis), congenital malformations, and cochlear otosclerosis [6].

The World Health Organization (WHO) classifies HL into four main grades based on the severity of hearing impairment, measured in decibels (dB) [11]:

- Mild HL (26–40 dB): Individuals can hear speech at a normal volume from about one meter away but may struggle with softer sounds, such as whispers or quiet conversations. Understanding speech in noisy environments can also be challenging.
- Moderate HL (41–60 dB): Speech must be spoken at a raised volume from one meter away for individuals to hear clearly. Normal conversations, particularly in the presence of background noise, may be difficult to follow.
- Severe HL (61–80 dB): Individuals can only hear a few words spoken at a high volume and experience significant difficulty perceiving speech at normal levels.
- Profound HL (>80 dB): Speech is inaudible, even when spoken loudly.

The term “deafness” is more accurately used to describe profound HL that significantly affects language access, particularly if not properly managed during early childhood (Table 1). A Deaf individual experiences total or near-total HL, making it impossible to clearly perceive sounds and spoken language without appropriate hearing devices.

### 2.2. Epidemiology, Impact on Quality of Life, and Treatments

Approximately 430 million people worldwide experience disabling HL, including more than 34 million children, and require rehabilitative interventions [12]. “Disabling HL” is defined as a hearing reduction greater than 40 dB in the better-hearing ear for adults and greater than 30 dB in the better-hearing ear for children [11] (Table 1). It is estimated that by 2050 approximately 2.5 billion people worldwide will have some form of HL, with at least 700 million requiring hearing rehabilitation [12]. These data highlight a worrisome trend driven by factors such as population aging, exposure to loud noise, and the impact of severe middle ear infections. In Western countries, the prevalence of congenital HL is estimated at 1 to 3 newborns per 1000 live births, with a significantly higher rate (32 per 1000) among infants admitted to neonatal intensive care units [13]. When left untreated, HL can significantly impact various aspects of individual and collective life, including the following [5,12,14,15,16,17,18,19,20]:

- Communication and language: Difficulty in hearing can hinder language acquisition and development, especially in young children, impairing their ability to communicate effectively with others.
- Cognitive abilities: The impact of HL on cognitive function differs across the life span. In children, uncorrected HL can negatively affect cognitive development, particularly interfering with learning processes and responsiveness to environmental stimuli. In older adults, age-related HL is one of the most significant modifiable risk factors for cognitive decline and dementia. Several mechanisms have been proposed to explain this link, including common neuropathology affecting both the cochlea and cortical regions, a reduced cognitive reserve due to sensory deprivation, an increased cognitive load from effortful listening, and interactions between brain activity related to auditory cognition and dementia pathology. Recent evidence suggests that the appropriate treatment of HL can delay the onset of dementia in a substantial proportion of the elderly population.
- Social interactions: Hearing difficulties often lead to social isolation, worsened by the inability to understand what others are saying. This isolation can result in loneliness, anxiety, and stigmatization, preventing individuals with HL from fully participating in social life.
- Economic burden: According to the WHO, the global annual cost of untreated HL is estimated at approximately USD 980 billion. This figure encompasses healthcare expenses (excluding hearing aids), educational support, lost productivity, and social costs. Low- and middle-income countries bear the heaviest burden due to limited resources for addressing and managing HL.
- Education and employment: In low- and middle-income countries, children with HL often lack access to adequate education. Adults with hearing deficits also face challenges in entering the workforce, with higher unemployment rates compared to the general population. Moreover, those who are employed tend to hold lower-level positions than their peers without hearing impairments.

The temporal trends between 1990 and 2019 highlight that HL has become an increasing source of disability in the United States of America (USA), with a particularly significant impact on the elderly. Hearing impairment is the most common sensory disorder and ranks as the third leading cause of disability in individuals over 70 years of age. This trend is expected to escalate further as the population ages, making HL management an increasingly urgent public health priority [21].

From a clinical perspective, in cases of congenital Deafness, hearing aids should be fitted within the first four months of life, ideally as soon as severe or profound bilateral sensorineural HL is confirmed [22]. Pediatric hearing aid fitting is a complex process requiring collaboration among otolaryngologists, audiologists, and speech therapists. Early diagnosis, followed by appropriate hearing aid fitting and speech therapy, is essential for optimal outcomes within the medical model. Similarly, acquired HL should be promptly treated with hearing aids to prevent the deterioration of language skills [12]. Digital hearing aids consist of a microphone, an analog-to-digital converter, an amplifier, a digital signal processor, a digital-to-analog converter, a receiver (or speaker), and a battery. The microphone captures sounds from the surrounding environment and converts them into an analog (electrical) signal. This analog signal is then sent to the analog-to-digital converter, which transforms it into a digital format (a series of numbers). The amplifier increases the intensity of the digital signal before it undergoes processing. The digital signal processor performs advanced sound processing, such as reducing background noise, enhancing specific sound frequencies (e.g., to improve speech comprehension), and adjusting the sound based on the environment (e.g., optimizing listening in a noisy restaurant). After processing, the signal must be converted back into an analog format to be heard. This is performed by the digital-to-analog converter. Finally, the receiver (or speaker) delivers the sound into the person’s ear canal. The battery powers all the electronic components of the hearing aid and may be rechargeable or disposable, depending on the device model. Based on their size and placement in the ear, hearing aids are classified into four main types: Behind-The-Ear (BTE), Receiver-In-Canal (RIC), In-The-Canal (ITC), and In-The-Ear (ITE) [23].

It is important to emphasize that in cases of profound bilateral HL, conventional hearing aids are generally not sufficient to ensure adequate auditory perception. In such situations, cochlear implantation is required. Unlike hearing aids, which amplify sound and stimulate the residual cochlear function, a cochlear implant directly transmits electrical impulses to the auditory nerve fibers via an electrode array surgically inserted into the cochlea (Table 1). This process bypasses the non-functioning cochlear receptors, effectively making the cochlear implant an “artificial cochlea” [24]. The optimal age for cochlear implantation is typically around 12 months, as this age offers the best risk–benefit balance, promoting optimal auditory development. However, in preterm infants, the procedure is usually postponed until 18–24 months to allow for the complete maturation of the auditory pathways [13]. In cases where Deafness is caused by meningitis, the risk of cochlear ossification increases, potentially hindering electrode insertion. In such circumstances, early implantation—even before the infant reaches one year of age—is recommended to maximize the chances of auditory recovery [24].

Contraindications to cochlear implantation include the following [25,26]:

- The agenesis of the auditory nerve: In the absence of the auditory nerve, a cochlear implant cannot transmit electrical impulses to the central nervous system, rendering the procedure ineffective.
- Cochlear aplasia: When the cochlea is completely absent, the implant cannot be inserted as there is no structure to house the electrodes.
- A lack of auditory memory: If an individual has never used hearing aids and has not developed auditory memory, a cochlear implant may not be effective. Auditory memory is crucial for rehabilitation, allowing the brain to recognize and interpret sound signals. Additionally, neuroplasticity—the brain’s ability to adapt to new stimuli—is particularly high in early childhood but significantly declines after the age of seven. This factor is critical in cochlear implantation, as late intervention following prolonged auditory deprivation may not be as effective as implantation during early childhood, when the brain remains highly adaptable to new stimuli.

When cochlear implantation is not feasible, alternative communication methods continue to play a fundamental role. The use of sign language, in particular, provides a visually based mode of interaction that does not depend on auditory perception, facilitating communication and improving the quality of life for individuals who are not eligible for surgical procedures [27].

In addition to these established treatments, recent advances in gene therapy have introduced promising new options. Notably, children with Deafness due to otoferlin mutations have shown the potential to regain their hearing through gene therapy, as demonstrated in a recent study by Wang et al. [28]. This breakthrough marks a significant step forward in the treatment of genetic forms of HL and may pave the way for future curative approaches.

## 3. Psychological, Gender, Cultural, and Social Aspects Related to Hearing Loss

### 3.1. Social Stigma

The stigma associated with HL and the use of hearing aids remains a widespread issue [29]. As the sociologist Erving Goffman highlighted, stigmatized individuals are those who exhibit physical or behavioral characteristics considered “deviant” or “abnormal” compared to a reference group in a given society [30]. Historically, prejudices against individuals with HL have had deep roots. In ancient Greece, Deaf individuals were deemed irrational due to the belief that thought required speech. The Romans, embracing the Greek ideal of physical perfection, subjected Deaf infants to cruel practices. Unfortunately, some of these biases persist today, significantly impacting the inclusion and well-being of those experiencing HL [31]. Misconceptions include the belief that raising a child with a disability is harmful to other children’s development, as well as the notion that individuals with disabilities are entirely dependent on government assistance and unable to contribute to society. These prejudices create social barriers and limit opportunities for meaningful integration [32]. Particularly, HL is often perceived as a sign of aging or vulnerability, reinforcing the notion that those affected are “different” or “inferior” to others [29,33]. As a result, the use of hearing aids is frequently seen—at any age—as an admission of weakness, leading many individuals to avoid wearing them out of a fear of judgment or negative labeling. Stigma primarily manifests as feelings of shame, anxiety over social rejection, and concerns about appearances [34]. Indeed, only a small percentage of individuals who could benefit from hearing aids use them, with estimates ranging from 16% to 30% [35]. There is a common misconception that hearing aid use in children is linked to poor health, low confidence, limited athleticism, and decreased popularity or leadership. This negative perception can discourage children from wearing hearing aids and experiencing their full advantages. To combat this, it is crucial to promote the visibility and positive representation of hearing aids, showcasing them as empowering tools that support communication, confidence, and success [36]. It is interesting to note the significant difference in social perceptions between eyeglasses and hearing aids. Once considered a sign of visual weakness and vulnerability, eyeglasses have overcome these prejudices through a significant evolution in design, transforming from a medical necessity into fashionable accessories. This shift was supported by the introduction of new features, such as in sunglasses and personalized models, and the influence of famous actors and singers, who helped make them more accepted and desirable [37]. A similar transformation could occur with hearing aids, which are evolving in both design and functionality to meet increasingly diverse needs and consumer expectations [38]. Beyond simple sound amplification, modern hearing aids may gain greater acceptance through the incorporation of advanced features, such as wireless connectivity with mobile devices, background noise reduction, and automatic adaptation to different acoustic environments. As their design and performance continue to improve, these advancements in hearing aids could help reduce the stigma perceived by users [37,38]. In recent years, cochlear implants have also undergone miniaturization and technological advancements. Traditionally, the external component of a cochlear implant included a microphone, a speech processor, and a battery, all positioned behind the ear and connected to a transmitter fixed using a magnet onto the outer surface of the squamous part of the temporal bone. Recently, “all-in-one” audio processors have been developed, integrating all the components into a single and compact device worn outside the ear, typically on the temporal area. Examples include the Cochlear Kanso™ and the MED-EL Rondo™. These innovative devices are valued for their discreet design and comfort, as they reduce visibility and enhance the user experience, making cochlear implants feel less intrusive [39].

In promoting social acceptance, the animated film “Toy Story 4”, produced by Pixar Animation Studios and released in June 2019, played a unique role by introducing a new character: a child with a cochlear implant. This character stands out for two key reasons: he is fully integrated into the social setting and is shown smiling. Inclusivity and joy are often missing in media portrayals of individuals with HL, but the presence of this happy character has had a meaningful impact in challenging negative stereotypes and fostering a more balanced perception of hearing impairment [40]. In addition, in June 2022, Mattel released a Barbie doll featuring pink BTE hearing aids, developed in collaboration with Dr. Jen Richardson, an experienced audiologist, to ensure realistic representation. This doll not only allows children with HL to see themselves represented but also fosters awareness and empathy, helping to normalize differences from an early age [41]. As a matter of fact, overcoming the stigma associated with HL requires a collective effort that combines awareness, education, and cultural change. It is essential to promote informational campaigns on HL that explain the symptoms, causes, and available solutions, such as hearing aids, to reduce fear and ignorance [38]. Additionally, widespread accurate and positive representations of people with HL in films, TV shows, social media, music, and books could further support the process of social acceptance.

### 3.2. Gender Differences

Women with HL face multifaceted challenges that often exceed those encountered by other groups with disabilities. As early as the 15th century, the Spanish nun Teresa de Cartagena, who became Deaf due to illness, addressed these issues in “Admiraçión operum Dey”. Her work not only explored the societal role of women but also critiqued the entrenched gender discrimination of her era. Notably, she meticulously countered detractors who doubted that her earlier work, “Arboleda de los Enfermos”, could have been authored by a woman, let alone a Deaf one [42].

While individuals with disabilities already face considerable barriers, women confront additional hurdles in workforce entry due to deep-seated prejudices and cultural constraints [43]. In the USA, although the employment gap between men and women stands at 11.8% among hearing individuals and 10.6% among Deaf individuals, significant wage disparities persist. On average, Deaf women earn just USD 0.77 for every USD 1.00 earned by Deaf men, compared to USD 0.83 for hearing women relative to hearing men [44]. Addressing these inequalities requires targeted awareness policies to dismantle barriers to wage equity and cultivate inclusive workplaces where women with hearing disabilities can fully realize their potential with equal rights and opportunities. Additionally, the psychological burden of HL is often more pronounced in women than in men. Studies have indicated that women are more likely to conceal their hearing difficulties, often due to societal expectations that cast them as emotional anchors within the family [45]. Social beauty standards exacerbate this issue, as hearing aids and cochlear implants may be perceived as a detriment to an individual’s public image, particularly among younger women [46]. Social media intensifies these pressures by propagating unattainable beauty ideals, contributing to body dissatisfaction and negative mental health consequences such as anxiety and depression [47]. HL significantly impacts women’s daily lives, making interpersonal relationships more complex. Communication often feels like a demanding effort, leading to uncertainty, stress, and fatigue. As they age, the combined effects of hearing impairment, declining health, and other age-related challenges further intensify their daily struggles, adding to their emotional strain [45].

A particularly concerning yet underreported issue is violence against Deaf women. Studies have suggested that Deaf women are twice as likely to experience domestic violence as their hearing counterparts [48]. Perpetrators often exploit their victims’ disabilities to exert greater control, a phenomenon known as “hearing privilege” [49] (Table 1)**.** Tactics include withholding access to communication devices, manipulating authorities, and socially isolating victims by controlling their interactions with the hearing world [50]. In some cases, abusers physically target the hands and arms of Deaf women to prevent them from signing [51]. The limited availability of support and the challenges in accessing assistance services extend the duration of abuse, further deteriorating victims’ living conditions [52]. Alarmingly, many Deaf women do not realize they are experiencing gender-based violence and fail to recognize that the mistreatment they endure is a criminal act [49,53].

Despite some progress, significant efforts are still required to dismantle the structural and cultural barriers faced by women with HL. Comprehensive strategies must encompass legal protections, workplace inclusivity, mental health support, and enhanced accessibility to services for victims of violence. Public awareness campaigns and targeted policy actions remain essential to fostering a truly equitable society where Deaf women can thrive without discrimination or marginalization.

### 3.3. Cultural and Social Differences

Cultural differences related to Deafness are influenced by various factors, including the traditions, history, and social norms that shape the daily lives of Deaf individuals. These differences manifest both individually and collectively and can vary not only between countries but also within different groups and associations, which often develop their own distinct identities. A key distinction lies between “deaf oralists”, who prefer verbal communication and lip-reading, and “deaf signers”, who use sign language [54]. Globally, more than 300 sign languages, dialects, and other signed communication systems have been identified [55]. The medical model views Deafness as a disability to be corrected through medical interventions, such as cochlear implants. In contrast, the social model interprets the term Deaf (written with a capital “D”) as a cultural identity, framing membership of the Deaf community as a privilege (“Deaf gain”) rather than a disability [54]. The Deaf community has the highest rate of endogamy among all social groups, with estimates suggesting that over 90% of marriages occur within the community [56]. However, it is important to note that approximately 90% of Deaf children are born to hearing parents, meaning that the dominant language and culture in most cases remain those of the hearing world [57].

In 1975, Tom Humphries, an American academic, coined the term “audism” to describe the belief that individuals are superior based on their ability to hear or conform to auditory norms (Table 1). Audism manifests as prejudice and discrimination both from hearing individuals toward Deaf people and among Deaf individuals themselves. It is evident in the tendency to measure intelligence and success by proficiency in the dominant spoken language, coupled with the assumption that a Deaf person’s well-being depends on acquiring that language. Additionally, those who are more aligned with spoken language and hearing cultural norms may exert greater social influence, imposing these standards on others within the Deaf community [58].

In his book “Understanding Deaf Culture: In Search of Deafhood”, written in 2003, Paddy Ladd, a British Deaf scholar and activist, introduced the concept of “Deafhood”, which describes a conscious awareness of being Deaf and the societal recognition of this identity (Table 1). Deafhood involves embracing Deaf culture, including its traditions, values, customs, and sign language. This perspective contrasts with the medical model of Deafness, which frames it as a pathological condition requiring treatment. While Deafness is often clinically defined as a disability, Deafhood affirms and celebrates Deaf cultural identity, rejecting the notion that Deafness should be “normalized” through medical intervention [59]. From this perspective, treating Deafness as a problem to be solved is fundamentally at odds with the principles and beliefs of Deaf culture (Table 1). As a result, many Deaf individuals oppose cochlear implants, viewing them as a form of “cultural genocide” that threatens the survival of the Deaf community, its language, and its culture [56,60]. Key reasons for this opposition include the belief that Deafness does not require a cure, the rejection of the notion that Deaf people are inferior to hearing individuals, concerns over the medical risks associated with cochlear implantation, the inability of young children to provide informed consent, and the perception of the procedure as an ethical violation within the Deaf community [56,60,61]. Critics of this perspective argue that it is selfish and unethical for parents of Deaf children to reject cochlear implants solely to preserve their cultural identity. This debate is particularly evident online, where videos of children’s cochlear implant activations often receive widespread praise from viewers but also draw criticism from Deaf individuals. In turn, these Deaf critics frequently face harsh backlashes, highlighting the deep divisions surrounding this issue [62]. To address parental resistance, healthcare professionals must adopt effective communication strategies to engage with families, ensuring that decisions remain within the healthcare context and are not influenced by external pressures. It is crucial to respect sign language and bilingualism as viable communication options while also highlighting their benefits for both society and Deaf individuals. If a family refuses medical treatment necessary for a child’s well-being, the final decision may be made by a legal guardian, though this should be considered only as a last resort [63].

Beyond the decision to identify with the Deaf community or to forego cochlear implantation for a Deaf child, it is important to recognize that this surgical procedure is highly expensive. Without health insurance, the cost of a cochlear implant in the USA ranges from USD 40,000 to USD 60,000 [64]. As a result, the caregivers of Deaf children born in developing countries or to low-income families often cannot afford the surgery. These financial barriers contribute to the phenomenon of “medical tourism”, where individuals travel abroad to seek medical care. Initially, medical tourism primarily involved patients from less developed countries traveling to wealthier nations for specialized treatments unavailable locally. However, a gradual shift has occurred, with more patients from industrialized nations seeking medical care in lower-income countries due to significantly lower costs [65]. Regarding cochlear implants, individuals traveling from the USA to India typically face total costs ranging from USD 21,000 to USD 30,000, including for travel, surgery, and the device. Although this is considerably lower than the costs in the USA, the price difference is less pronounced compared to that in other medical specialties, such as cardiovascular and orthopedic surgery [64].

Another critical issue concerns disparities within the Deaf community itself. For example, studies have shown that Black Deaf individuals face both racism and audism, experiencing even greater discrimination and encountering additional barriers to education, employment, and healthcare [66]. Similarly, LGBTQIA+ (lesbian, gay, bisexual, transgender, queer, intersex, asexual, and other gender identities) Deaf individuals often encounter increased discrimination, victimization, and a lack of adequate social support. The resulting stress significantly increases their risk of physical and mental health issues, particularly among older adults [67,68]. When a person has multiple marginalized identities—such as being Deaf, Black, and transgender—they may experience chronic stress, compounded by prejudice and discrimination, making them more vulnerable to chronic illness [68]. Overall, LGBTQIA+ individuals with disabilities navigate heightened challenges across various aspects of life, including education, career opportunities, financial stability, healthcare access, and intimate relationships, despite legal protections against discrimination in Western regions [69].

In 2002, a significant ethical debate arose when a Deaf lesbian couple in the USA deliberately chose to have a Deaf child by using sperm from a donor with a family history of Deafness. Like other members of the Deaf community, they did not view Deafness as a disability but rather as an essential part of their cultural identity. This case highlighted the desire of some Deaf couples to have Deaf children, even turning to prenatal genetic testing and in vitro fertilization to increase the likelihood of this outcome, raising profound ethical and moral questions [70,71].

## 4. Accessibility for Individuals with Hearing Loss

### 4.1. Barriers to Accessibility

Accessibility refers to the design of products, services, environments, and technologies to ensure equal access and usability for all individuals, including those with disabilities [72]. Barriers to accessibility for people with HL can be classified into three main categories: communication barriers, architectural barriers, and technological barriers [73,74,75]. *Communication barriers* often result from a lack of written or visual information, as well as a gap in understanding between service providers and individuals with HL. This gap becomes evident when staff responsible for providing customer service and assistance do not employ effective communication techniques, such as speaking clearly and at an appropriate volume, ensuring proper vocal projection to facilitate comprehension [74,75]. In this context, the cultural and linguistic competencies of service providers play a crucial role in fostering full inclusion for people with HL, facilitating effective interactions. The goal is to create the necessary conditions for assisting and welcoming all individuals, regardless of the language they use, so that everyone can fully enjoy their experiences [76]. However, in many public spaces such as museums, theaters, parks, and trails, information is not sufficiently adapted to meet the needs of people with HL. For example, many attractions lack subtitles, multimedia guides, maps, brochures, and appropriate signage, thereby compromising accessibility [77,78]. Additionally, the absence of specific training for staff and a general lack of awareness regarding the needs of individuals with HL significantly hinder effective communication and inclusion. The distance from a guide, which makes lip-reading difficult, and the necessity of focusing on listening during guided experiences—thus limiting the ability to observe the surrounding environment—represent further obstacles to participation. Large outdoor spaces at historic sites and attractions can create insurmountable communication barriers, making it difficult to engage with guides or interact with other visitors. Moreover, Deaf visitors often struggle to access sign language interpreters, as these professionals are frequently unavailable or difficult to find in public facilities [73,75].

*Architectural barriers* for people with HL can be linked to various factors, including the acoustic conditions, lighting, signage, and overlapping sound sources. For those using hearing aids or cochlear implants, environments with poor acoustics or excessive background noise can be particularly challenging [77]. The lack of adequate amplification devices, such as hearing loops or microphones, further complicates comprehension [79]. Ticket offices at various attractions, often equipped with partitions separating staff from visitors, can create significant communication difficulties. This hindrance can generate frustration and anxiety for individuals with HL, negatively impacting their overall experience from the outset. Furthermore, in situations where lip-reading is essential, poor lighting can cause considerable discomfort. In particular, guided tours in enclosed spaces such as museums and theaters, where lighting conditions are suboptimal, can severely hinder the ability of Deaf individuals to follow spoken information [80]. A significant obstacle is also the inadequate communication in public infrastructure, such as airports and railway stations, as well as public transportation systems like those for buses and trains. Audio announcements that convey essential information are often not transcribed into text or displayed on screens, which impedes access for Deaf people [74,81]. In emergency situations, the absence of visual or tactile signals can be extremely dangerous, as it prevents Deaf individuals from promptly receiving vital messages. The lack of alternative communication methods, such as the full conversion of verbal information into written text or sign language, increases the risk of disorientation or a failure to respond in time, thereby endangering their safety [78]. In crowded or noisy environments, the situation becomes even more complex. The overlapping of various sounds can be particularly problematic for individuals with HL, as it makes it difficult to focus on specific sources of information. For example, simultaneous background music, conversations, voice announcements, and environmental noises can obscure essential messages. Without appropriate technical solutions, an excessively noisy environment not only limits accessibility but also creates disorientation, compromising the safety and well-being of Deaf individuals [80,82]. Indeed, the architectural challenges for Deaf people are numerous, and mitigating them requires careful attention to detail. One of the main difficulties is the budgetary constraints and limited resources that prevent the achievement of the desired level of accessibility [83]. Another obstacle is the lack of awareness among architects regarding the specific needs of the Deaf community. Designers often lack adequate knowledge of accessibility principles, which can result in spaces that are not sufficiently inclusive or inadvertently exclude individuals with hearing impairments. Moreover, while people with HL require spaces that facilitate visual communication, esthetic priorities sometimes take precedence over practicality, neglecting accessibility considerations [83,84].

*Technological barriers* to accessibility for individuals with HL primarily involve the limited availability of assistive technologies that could enhance participation in everyday activities. These barriers go beyond translation devices or visual alert systems and include assistive technologies that are not widely implemented or accessible in many public spaces, such as frequency modulation (FM) systems, hearing loops, and speech recognition software [79,85,86,87]. Indeed, the high costs associated with these technologies hinder their adoption, particularly in countries with limited economic resources. Moreover, regulatory disparities across countries affect the widespread implementation of assistive technologies, while inadequate infrastructure in certain areas further restricts their deployment [85,88]. The development and adoption of assistive technologies are also shaped by ethical concerns, including data privacy, security, and the societal perceptions of individuals with HL. Overcoming these challenges requires collaboration among researchers, industry experts, and end users to foster innovation and inclusivity [89]. One potential solution lies in the availability of “smart sensors”, which capture and process environmental information, such as sounds and noise, and convert it into perceivable signals for individuals with HL, such as vibrations, visual alerts, or other non-auditory notifications. However, the integration of these sensors remains limited, as many devices are custom-built or developed in research institutions using technologies that are not yet widely available on the market. Nevertheless, continuous technological advancements and growing demand are expanding the range of available sensors, paving the way for increasingly effective and accessible assistive solutions [87].

In this context, the sociologist Zygmunt Bauman emphasized how contemporary society constructs the notion of normality around the needs of the majority, thereby marginalizing individuals with hearing disabilities. Common tools such as telephones, doorbells, and auditory alerts—designed for collective use—become inaccessible to those with hearing impairments, a challenge Bauman personally faced after experiencing significant HL in his later years. This illustrates how societal structures are built around the needs of the “normative” majority, often overlooking those who do not conform to this standard. To dismantle such exclusionary barriers, Bauman advocated for an inclusive society that actively embraces accessible technologies and targeted policies to ensure equal dignity and opportunities for all [90].

### 4.2. Enhancing Accessibility: Key Considerations and Solutions

**Figure 1:**
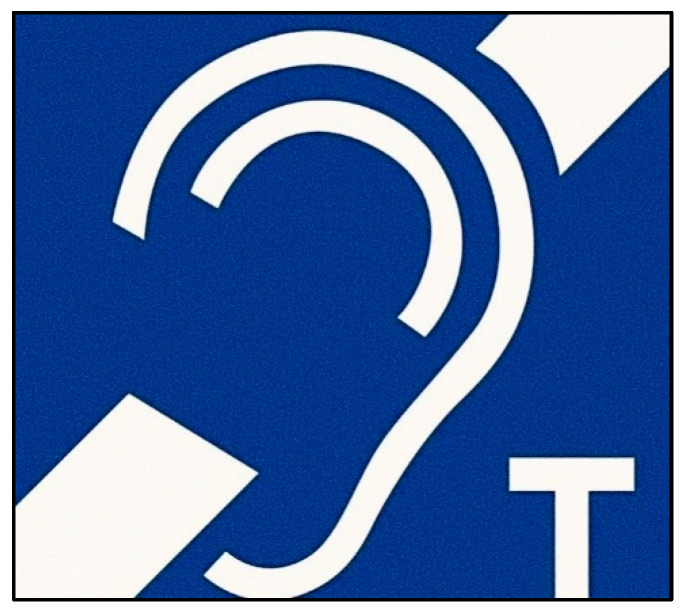
Internationally recognized hearing loop sign.

Ensuring accessibility for individuals with hearing disabilities presents an ongoing challenge that requires the implementation of specific measures to guarantee full and satisfying experiences in various aspects of daily life. Before arrival at any venue, it is essential to provide comprehensive and reassuring information to eliminate uncertainties or concerns. Websites, as the primary point of contact, must be designed to be accessible and inclusive, using simple and clear language balanced with visual content. Moreover, they must include text transcriptions and subtitles for audio and video content, and the navigation should be intuitive to ensure full comprehension. In addition to phone contact, alternative methods such as email, online chats, and social media must be available to facilitate communication and streamline the booking process for individuals with HL [91]. Training and education for employees in public-facing roles are crucial. Proper training on how to interact with individuals who are Deaf or hard of hearing can significantly improve the accessibility and inclusivity of a service. For example, it is important to use respectful and appropriate language, avoiding outdated terms such as “invalid” or “deaf-mute” and instead using expressions like “person with hearing loss” or “deaf person”. Additionally, raising awareness among staff about effective communication techniques is key for facilitating interaction. These include using short and simple yet complete sentences, making sure the speaker’s lips are clearly visible, and maintaining a clear and well-modulated tone of voice [77]. Another useful strategy is to attract attention before speaking by using visual signals or gestures. This enables individuals with HL to anticipate the information, minimizing the risk of a misunderstanding or interruption [92]. For people who are Deaf, using sign language interpreters can improve their access to information and create a more inclusive experience [91]. Along with alternatives to verbal communication, the use of assistive technologies is essential for improving accessibility for individuals with HL. Sound amplification devices, such as headphones or FM systems, are particularly useful as they allow individuals to hear clearly, even in noisy environments [93]. A noteworthy technology is a hearing loop system (Table 1): Sound is captured by a microphone, converted into a magnetic signal via an amplifier, and transmitted to individuals with HL through an audio induction loop. The magnetic signal is then received and converted back into audible sound by a telecoil (a telephone coil) in hearing devices, such as hearing aids or cochlear implants set to the T (telecoil) mode. These magnetic induction systems, managed using a specific amplifier (loop driver), wirelessly transmit signals directly to hearing devices, improving the communication quality and reducing environmental noise [79,93]. The benefits of hearing loops are numerous, and they represent an innovative solution to enhance auditory accessibility in various public spaces. Firstly, these systems can be used by anyone with a compatible hearing aid or cochlear implant without the need for additional headphones or receivers, as the sound is transmitted directly to the hearing device. Additionally, there is no limit to the number of individuals who can simultaneously benefit from the system, making it especially valuable in crowded spaces. Another significant advantage is their ability to eliminate background noise, enhancing message clarity [94]. As a result, the adoption of hearing loop systems in areas like service counters, reception desks, meeting rooms, theaters, cinemas, museums, waiting areas, public transport, and assistance points significantly enhances the participation and social engagement of individuals with HL. The sign indicating the presence of a hearing loop system is internationally recognized, ensuring that people can easily identify its location and usage [93,94] (Figure 1). For example, in New York, hearing loop technology is widely implemented. Public transportation has been equipped with advanced hearing loop systems to improve accessibility: all information desks in subway stations feature this technology, facilitating interaction with passengers. Similarly, more than 2800 “Taxis of Tomorrow” have been designed to include hearing loops, allowing users with hearing aids or cochlear implants to communicate with drivers directly and clearly [95]. Furthermore, the integration of QR codes for accessing information via a video or text, the incorporation of augmented reality experiences to provide additional details, and the development of interactive displays with visual content can significantly improve communication clarity and effectiveness [77,78]. Visual aids help overcome barriers caused by the inability to perceive auditory signals. These tools, which can benefit both individuals with and without hearing impairments, include visual alerts, illuminated indicators, traffic lights with visual danger warnings, and continuously active monitors. Implementing such technologies in high-traffic areas such as train stations, airports, and museums can greatly improve accessibility [96]. In South Korea, three interactive prototypes have been developed to improve access to museum information, as traditional text-based descriptions often prove ineffective for individuals with HL. The “Active-linked model” features highlighted words that users can click on for additional explanations, enhancing comprehension without oversimplifying the content. The “Graph-based model” organizes information into an interconnected network, allowing users to explore concepts progressively, reducing cognitive overload while enabling the in-depth exploration of topics of interest. The “Chatbot-based model” employs an interactive chatbot to answer user inquiries, offering a more personalized and dynamic experience [97].

Some emerging technological advancements have built upon existing applications, such as smart glasses equipped with sign language interpretation. These devices utilize applications to translate spoken language into sign language, projecting the interpreter directly onto the lenses. Additionally, certain applications provide real-time captions, automatically transcribing conversations onto the glasses’ display. Further innovations include software designed for laptops and tablets, along with a variety of smartphone applications that enhance interaction and accessibility [89,98,99]. Applications designed for individuals who are Deaf or hard of hearing must feature a clear and user-friendly interface, ensuring seamless navigation through different sections and functionalities. The layout should be simple and intuitive, with well-positioned interactive options and customizable features that facilitate effortless navigation and learning [99]. Research on technologies for individuals with HL largely aligns with mainstream technological advancements, as the digital revolution has prioritized visual communication. This has enabled the Deaf community to become early adopters of innovations such as video calls and instant messaging services. Currently, major developments are focusing on refining sensory substitution technologies, including vibrotactile and visual–auditory systems, which seek to replicate auditory experiences through alternative sensory channels [89,100].

Public spaces should cater to the needs of individuals with HL, addressing various factors that enhance mobility and communication [96]. Surfaces should be even and free of level changes, as HL is sometimes associated with balance issues. The environment should be free of blind spots to ensure full visibility and spatial awareness. The layout should incorporate designated pathways to guide individuals within spaces, improving visibility, particularly for those who rely on visual cues. A minimalist design, with fewer walls, is recommended to maintain a wide and unobstructed field of vision, using glass instead of partitions to separate spaces [101,102]. Lighting is another crucial element: proper illumination helps compensate for the inability to perceive auditory cues, allowing individuals to detect movement through visual contrasts. Both natural and artificial lighting should ensure clear and comfortable visibility while preventing glare. Lighting must also be uniform and stable to avoid sudden changes that could cause discomfort or disorientation [101,103].

The design of inclusive public buildings for individuals with HL also requires the careful consideration of noise management. Sound-absorbing panels, made from materials such as mineral fibers, foams, or technical fabrics, are designed to absorb sound energy, reducing echoes and reverberation. Beyond their acoustic function, these panels can be customized in terms of their shape, color, and style to integrate harmoniously into the esthetic of the environment. Laminated glass with an intermediate layer of sound-insulating material, such as polyvinyl butyral (PVB), represents an innovative solution for reducing sound transmission without compromising natural lighting. Acoustic sealants, composed of elastomeric materials, create airtight barriers that prevent the propagation of low-frequency noises through small openings [101,104]. Regarding interior design, several solutions can improve accessibility for individuals with HL. Door placement should avoid obstructing visibility, mirrors can be strategically positioned to expand the visual field, and spatial organization should facilitate active participation in interactions. The use of round tables, movable chairs, and open spaces enhances communication. Specific features recommended for public environments include podiums for sign language interpreters, screens for real-time captioning during events, and circular seating arrangements to foster inclusion and allow Deaf individuals to maintain visual awareness of their surroundings [101,105]. Directional signage should follow a logical sequence that clearly guides individuals with HL from their starting point to their destination. It is advisable to use bright, contrasting colors, well-spaced text, and highly legible fonts against distinct backgrounds, complemented by pictograms and schematic representations that are easy to interpret. Identification signage should clearly indicate the function and location of different spaces, allowing for intuitive navigation [77,101]. Vibrations provide an additional way to perceive and interact with the environment, particularly for individuals with HL. In this context, vibrations refer to tactile feedback—physical movements or oscillations transmitted through surfaces or objects—that serve as sensory substitutes for auditory cues, enabling the detection of events or interactions that might otherwise go unnoticed. In inclusive design, it is essential to distinguish between beneficial and disruptive vibrations. Beneficial vibrations can facilitate communication through subtle contact with floors or furniture in designated areas. Conversely, unwanted vibrations from mechanical equipment or foot traffic must be minimized to prevent distractions and interference with important signals. Material selection is key—some surfaces should enhance useful vibrations, while others should dampen unnecessary ones. Special attention should be given to creating “vibration zones” that act as sensory thresholds between public and private spaces. These zones discreetly signal the presence of visitors or nearby movement, enabling smooth transitions and immediate awareness without the need for vocal cues [101,105].

Ultimately, accessibility for individuals with HL relies on a coordinated approach that includes staff training, assistive technologies, and inclusive design. By implementing these measures, environments can become more inclusive, allowing everyone to participate fully and independently.

## 5. Conclusions

Providing accessibility for individuals with HL is a crucial step toward building a more inclusive and equitable society. While technological and medical advancements—such as cochlear implants and modern hearing aids—have transformed the landscape of hearing impairment, significant barriers remain, both cultural and structural, that limit full and independent participation in various aspects of life. A key consideration is the evolving characteristics and needs of individuals with hearing disabilities. The number of newborns who rely solely on sign language is steadily decreasing due to the introduction of cochlear implants. However, a rising life expectancy and increasing noise pollution are contributing to a growing number of individuals with HL. This shift necessitates a reevaluation of accessibility strategies, extending the focus beyond sign language interpreters to include solutions that enhance the experience for individuals using hearing aids or cochlear implants. In this scenario, it is essential to implement measures such as noise reduction systems in public spaces, transportation, and accommodation to improve auditory perception and reduce acoustic overload. Advanced assistive technologies, such as sound amplifiers and induction loop systems compatible with hearing aids, can enhance the auditory experience in everyday situations. Another key aspect is the necessity of clear and visible signage, supplemented by pictograms and textual instructions, to facilitate the easier navigation of spaces. Subtitling audiovisual content, from multimedia guides in museums to informational videos in airports and in transportation systems, serves as an additional tool for inclusion. Lastly, training public-facing staff is vital for enhancing hospitality and communication, thereby fostering a more inclusive environment.

However, it is important to emphasize that improving accessibility for individuals with hearing disabilities cannot be limited to technical or structural interventions. A cultural shift is essential to dismantle the stereotypes that still persist in society. Deafness is often accompanied by stigma, which manifests in various forms, from the false belief that a Deaf person is automatically incapable of communicating or understanding to gender discrimination, which causes many women to feel uncomfortable using hearing aids for fear of being judged. Regarding the use of hearing aids and cochlear implants, it should be noted that, especially in low-income or developing countries, economic inequalities and limited access to healthcare technologies prevent a significant portion of the population from benefiting from these devices. The future of accessibility should focus on cultural change through awareness campaigns, promoting positive experiences, and actively involving individuals with HL in the design of services and spaces. Only in this way can we shift from a model “for the few” to a truly inclusive one where every individual, regardless of their sensory abilities, can fully engage in activities, explore new environments, and enjoy experiences without barriers. Investing in accessibility is not only a matter of equity and social responsibility but also an opportunity to enhance participation for a growing demographic. A collaborative effort between institutions, service providers, and the broader community is crucial for building an inclusive society where individuals of all hearing abilities can thrive and participate without limitations.

## Figures and Tables

**Table 1:** Key terms and concepts related to hearing disability.

Terms/Concepts	Definition
Audism	A form of discrimination that assumes individuals who can hear or conform to auditory norms are superior to those who are Deaf, leading to social exclusion.
Cochlear implant	A surgically implanted electronic device that provides auditory stimulation to individuals with severe to profound sensorineural hearing loss, helping them perceive sound.
Deaf culture	A linguistic and social community centered around sign language, shared experiences, and collective identity, recognizing Deafness as a cultural gain rather than a condition to be corrected.
Deafhood	A self-actualized identity that embraces Deafness as a cultural and linguistic experience rather than a medical condition, recognizing the collective history and values of the Deaf community.
Deafness	A profound hearing loss that significantly affects language acquisition and communication, particularly if not addressed in early childhood.
Disabling hearing loss	A hearing reduction exceeding 40 dB in the better-hearing ear for adults and over 30 dB in the better-hearing ear for children.
Hearing aid	A small, wearable electronic device that amplifies sound for individuals with mild to severe hearing loss, enhancing their ability to communicate.
Hearing loops	Assistive listening systems that transmit audio signals directly to telecoil-equipped hearing aids and cochlear implants, improving accessibility and speech clarity in public settings.
Hearing loss	A reduction in auditory function that varies in type (conductive, sensorineural, or mixed) and severity (mild to profound), potentially affecting communication, cognitive development, and quality of life.
Hearing privilege	The advantages enjoyed by hearing individuals, including unrestricted access to communication, education, and safety. Deaf women face heightened risks of violence and abuse, as hearing perpetrators exploit communication barriers and the limited access to support services.

## Data Availability

No new data were created or analyzed in this study. Data sharing is not applicable to this article.
